# Measuring Public Reaction to Violence Against Doctors in China: Interrupted Time Series Analysis of Media Reports

**DOI:** 10.2196/19651

**Published:** 2021-02-16

**Authors:** Qian Yang, Ming Tai-Seale, Stephanie Liu, Yi Shen, Xiaobin Zhang, Xiaohua Xiao, Kejun Zhang

**Affiliations:** 1 Center for Health Policy Studies School of Public Health and Department of Endocrinology, Children's Hospital, Zhejiang University School of Medicine National Clinical Research Center for Child Health Hangzhou China; 2 Department of Family Medicine, School of Medicine University of California San Diego San Diego, CA United States; 3 School of Public Health Zhejiang University Hangzhou China; 4 College of Computer Science and Technology Zhejiang University Hangzhou China

**Keywords:** violence against doctors, government intervention, public opinion, patient–physician relationship

## Abstract

**Background:**

Violence against doctors in China is a serious problem that has attracted attention from both domestic and international media.

**Objective:**

This study investigates readers’ responses to media reports on violence against doctors to identify attitudes toward perpetrators and physicians and examine if such trends are influenced by national policies.

**Methods:**

We searched 17 Chinese violence against doctors reports in international media sources from 2011 to 2020. We then tracked back the original reports and web crawled the 19,220 comments in China. To ascertain the possible turning point of public opinion, we searched violence against doctors–related policies from Tsinghua University ipolicy database from 2011 to 2020, and found 19 policies enacted by the Chinese central government aimed at alleviating the intense patient–physician relationship. We then conducted a series of interrupted time series analyses to examine the influence of these policies on public sentiment toward violence against doctors over time.

**Results:**

The interrupted time series analysis (ITSA) showed that the change in public sentiment toward violence against doctors reports was temporally associated with government interventions. The declarations of 10 of the public policies were followed by increases in the proportion of online public opinion in support of doctors (average slope changes of 0.010, *P*<.05). A decline in the proportion of online public opinion that blamed doctors (average level change of –0.784, *P*<.05) followed the declaration of 3 policies.

**Conclusions:**

The government’s administrative interventions effectively shaped public opinion but only temporarily. Continued public policy interventions are needed to sustain the reduction of hostility toward medical doctors.

## Introduction

With the improvement of Chinese people's living standard [1] and the attendant increase in personal wealth, individual Chinese citizens are paying more attention to their quality of life and health [2]. Health concerns have gradually become the focus of public discourse and are among the most popular topics on the internet in China. Concurrent with this trend, the plight of the strained doctor–patient relationship has become more evident, with violence against doctors, nurses, and other health care providers in China on the rise [3]. Incidents of violence against doctors increased from 20.6 incidents per hospital in 2008 to 27.3 in 2012 [4]. An editorial in *The Lancet* in 2012 called this situation a “crisis” for the Chinese medical field [5].

In this period of mass media, online commentary and discussion have increasingly been proven as a useful instrument for surveying the moods and attitudes of the general public. The prevalence of the media and the internet for use in health communication has already become standard in regions such as China/Taiwan, where the media plays a significant role in settling medical disputes and dealing with medical institutions for patients [2]. For instance, the online community may share viewpoints on an individual physician or medical facility, which considerably shapes the image and evaluation of the physician or medical facility for the general reading public [6]. Moreover, those with media access that can further express their thoughts through media outlets can affect how news is portrayed and its content [7].

Public interest in the doctor–patient relationship and the widespread use of the internet have made it relatively easy for violence against doctors events to trigger a dispute of public opinion online. It has been suggested that the rise of online public discourse on violence against doctors may negatively affect the doctor–patient relationship and exacerbate the perception of social chaos and loss of control [8]. While many may agree that public opinions online are symbolic of challenges in real life, as reflected by online expressions of attitudes, views, and emotions, their impact reaches beyond the virtual world. Many studies have shown that online public opinion is relatively consistent in its intensity and continuity, influencing uncivilized network behaviors and social uncertainty [9,10].

To maintain social stability both online and offline, the Chinese government has made great efforts to regulate online content. For example, it is illegal to create or spread rumors on the internet in China as it may disrupt public order and social security [11]. In addition, the government has implemented a series of policies to increase the punishment for the crime of violence against doctors, and a government bureau, the National Radio and Television Administration, has published a series of articles to guide public opinion. Moreover, in July 2014, the National Health and Family Planning Commission, Ministry of Justice, Ministry of Finance, China Insurance Regulatory Commission, and National Administration of Traditional Chinese Medicine issued a vital policy—Opinions of the National Health and Family Planning Commission, the Ministry of Justice, the Ministry of Finance and Other Departments on Strengthening the Work of Medical Liability Insurance [12]—to establish a medical risk-sharing mechanism with medical liability insurance as the primary form and make this mechanism essential in medical dispute resolution and medical risk management. The Chinese Medical Association has issued a statement calling for system-wide reforms. In October 2013, the Ministry of Public Security of the People’s Republic of China advised hospitals with over 2000 beds to hire “at least 100 security guards” [13]. However, increased security guards, metal detectors, and legal threats have been criticized for failing to deal with the underlying causes of the violence [14].

These policies have been attributed to some reduction in the acceleration of violence against doctors and calming of public opinion on doctor–patient conflicts [15]. Patients feel a lack of control due to the uncertainty of illness [16,17]. Many people require personal control over their lives and the surrounding environment, perhaps to avoid feeling the chaos and uncertainty in the world [18,19]. When people feel lower levels of personal control, they are more likely to show more support for government control [20].

However, whether such policies and interventions have made a practical positive impact on online public opinion has not been examined. One way to investigate this question is to compare the timeline of a turning point in public comments and the timing of the introduction of violence against doctors–related policies. Comments of the article instead of its content could provide more intuitive reflection of the patients side [21]. Accordingly, to gauge the overall public attitude toward doctors and the health care system in China, we endeavored to measure online reactions to mass media reports of violence against doctors.

Comments on violence against doctors incidents expose the attitude of the affected party groups to a certain extent. In recent years, an increasing number of violence against doctors events have affected people’s perceptions of the country in a period of social transition, where intense emotions and perceptions of people and affected groups are also influenced by perceived sense of control [22], and government policy interventions can provide a compensatory sense of said control [20]. Therefore, to understand the emotional valence (sentimental valence) of public comment on violence against doctors episodes, it is meaningful to check whether national policy has affected the stereotypes associated with the doctor–patient bond from a time perspective.

To assess the effectiveness of government interventions in influencing online public opinion in China, we retrieved significant media reports of violence against doctors events and time-relative related government policies (eg, Criminal law or rules and regulations of the National Health Commission) in a policy database from 2011 to 2020 [12,15,23,24]. We documented public attitudes by coding comments on online media. Overall, we expected that these data would provide a better understanding of the general public who utilize social media and the internet, as well as insight into their attitudes toward health, perceived quality of health care services, and health care information needs. Thus, our research questions were (1) what trends in attitudes do the readers show with respect to the actions of both patients and physicians? (2) Are these trends influenced by the introduction and direction of national policies?

## Methods

### Sampling and Data Collection

To identify which events are internationally influential, we visited well-known media sites, including the Wall Street Journal, New York Times, BBC, Economist, Washington Post, Telegraph, Times, New Yorker, South China Morning Post, International Business Times, National Public Radio, and The Atlantic and found a total of 17 medical violence incidents reported in China between October 19, 2011, and April 4, 2020. In total, there were 29 English reports. We then traced these incidents to Chinese website media and crawled (using a web robot to collect scripts at high speed) them for encoding and analysis to estimate online public attitudes toward doctors and patients (Multimedia Appendix 1).

We selected popular Chinese news media (N=6) from the New Media Influence Index Report [25], and considered their lasting influence from 2011, as shown in Table 1. We selected these 6 news media for 2 reasons. First, when we traced back the Chinese violence against doctors reports in international media sources, these media had a higher report rate than other media [26]; second, we referred to the media influence from 2011 to 2019 and, according to the Kantar China Media Impact Report 2016 and 2019, these 6 news media were more influential during these years [27].

We obtained 17 cases and their comments from the selected news media (see Multimedia Appendix 2 for details). As 2 of the 17 cases occurred in private hospitals, pseudonyms were applied to both private and public hospitals. There were 3 second-class A hospitals, 1 second-class B hospital, 2 top-class B hospitals, and 9 top-class A hospitals. Thus, the study covers different levels of hospitals, consistent with the distribution of hospital ownership status and hospital level in the total sample of doctor–patient disputes [28]. As the median number of comments was 400 on each incident, based on the total media coverage, if the count exceeded 400, we took a random sample of 400. The comments of each case are arranged in chronological order and divided into X/2n areas, and 2 comments were randomly extracted from each area. If the count was under 400, all coverage was included in the analyses. We then eliminated the repeated and unrelated comments. If the number of meaningless comments in the selected final comment sample exceeded 20%, these comments were removed. The number of comments required was then supplemented by the above sampling method. The sampling method is based on a real-world situation. We then collated and analyzed all the comments we collected. The final comment numbers for each case are listed in Table 2.

**Table 1:** All news media used to obtain sample comments.

News media	Reference
Tencent News	[29]
Sohu News	[30]
iFeng News	[31]
Sina News	[32]
Tianya Forum	[33]
NetEase News	[34]

**Table 2:** Number of network reviews for all cases.

Case	2011 (N=255)	2012 (N=1245)	2013 (N=178)	2014 (N=1473)	2015 (N=889)	2016 (N=1003)	2018 (N=924)	2019 (N=721)	2020 (N=521)	Total (N=7209)
C1	254	428	1	3	—^a^	—	—	—	—	696
C2	—	384	—	5	—	—	—	—	—	389
C3	1	433	38	2	—	—	—	—	—	474
C4	—	—	139	474	247	—	—	—	—	860
C5	—	—	—	400	2	—	—	—	—	402
C6	—	—	—	189	—	—	—	—	—	189
C7	—	—	—	—	200	—	—	—	—	200
C8	—	—	—	—	158	—	—	—	—	158
C9	—	—	—	—	282	2	—	—	—	284
C10	—	—	—	400	—	—	—	—	—	400
C11	—	—	—	—	—	400	—	—	—	400
C12	—	—	—	—	—	400	—	—	—	400
C13	—	—	—	—	—	201	—	—	—	201
C14	—	—	—	—	—	—	396	3	121	520
C15	—	—	—	—	—	—	528	—	—	528
C16	—	—	—	—	—	—	—	539	—	539
C17	—	—	—	—	—	—	—	179	400	579

^a^“—” represents no count of the comments in the *given* year.

### Ethical Approval and Consent to Participate

Ethical approval was obtained from the School of Public Health, Zhejiang University. As open data obtained through web crawlers do not include any personal data, informed consent was not required (waived by the ethics committee), and the study involved minimal risk.

### Coding

#### Procedure and Steps

A coding group comprising 4 preventive medicine students was trained to analyze the coverages. The coding forms outlined 9 categories developed a priori based on previous literature [35,36] (Table 3). The coding was performed in MS Excel.

**Table 3:** Coding categories.

Category	Meaning/Definition
Blame Big System	The comments express dissatisfaction, anger, or blame for the whole society or social atmosphere.
Blame Medical System	The comments express dissatisfaction, anger, or blame for health policy or the hospital system.
Blame Doctor	The comments express dissatisfaction, anger, or blame for the doctor or his/her behavior, other doctors, or their behavior in the case.
Blame Patient	The comments express dissatisfaction, anger, or blame for the patient or his/her behavior, other patients, or their behavior in the case.
Blame Other	The comments express dissatisfaction, anger, or blame for other things, such as the news media, the legal system.
Support Doctor	The comments express understanding, sympathy, or support for the doctor or his/her behavior, other patients, or their behavior in the case.
Support Patient	The comments express understanding, sympathy, or support for the patient or his/her behavior, other patients, or their behavior in the case.
Support Other	The comments express understanding, sympathy, or support for other things.
N/A	Comments are unrelated to cases or do not belong to any of the above categories.

At the beginning of the evaluation, we trained the 4 raters in the method of “independent coding–proofreading–third party opinion discussion” and then ensured that the interrater reliability reached 0.9 or above before they began to evaluate the formal materials. All the formal comments assessments were completed according to the following steps.

#### Step 1

The coding team carried out training to define and unify the coding rules.

#### Step 2

Fifty sample comments were extracted, and each rater was asked to code these comments independently. We compared the coding results between raters to obtain the “interrater reliability” (interrater reliability = the number of entries with the same coding result/Total number of entries, ie, the consistency between all raters’ coding results). The final results were unified according to the coding rules.

#### Step 3

If interrater reliability was less than 0.9, we repeated the operations in step 2 until it reached 0.9. The coding team then began to code formal samples.

#### Step 4

The raters read and coded all the comments independently, without interfering or discussing with each other. Each comment could belong in 1 category or more.

#### Step 5

We compared the coding results of each rater to find comments that were inconsistent in the coding results.

#### Step 6

We established a discussion group to reread all the comments and to determine the coding results of comments that were inconsistent in step 4. Finally, the coding team completed the coding results of all the comments.

#### Step 7

For the comments between 2017 and 2020, we used a sentimental dictionary developed from the code between 2011 and 2016. The sentiment was Normalization as [0,1].

### Constructing a Sentiment Dictionary

We collected all the comments, marked each sentiment of each comment, and then segmented these comments into words, and removed some stop words to obtain the probability *P_ij_* of each word appearing in each sentiment:

*P_ij_*=(*e_j_*|*w_i_*) = x = [C(*w_i_*,*e_j_*)]/[C(*e*_j_)]

where *P_ij_* represents the probability that *w_i_* represents sentiment *e_j_*, and C(*w_i_*,*e_j_*) represents the number of times that *w_i_* appears in sentiment *e_j_*.

### Comment Sentiment Judgment

Thereafter, when judging new comments, we first segmented each comment and removed some stop words. According to the naïve Bayes principle, a certain sentiment of each comment is the accumulation of the sentiments of all participles of the comment. To eliminate the polarization problem, the calculation is multiplied by the value related to the length of each comment:

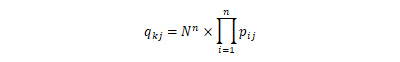



Finally, a threshold value is selected according to *q_kj_* to judge the sentiment determination.

### Policy Content Quantitative Analysis

Policy content quantitative analysis is a semantic analysis method that combines quantitative and qualitative analysis of the content of policy literature. A proper policy database could display the essential information of the content of violence against doctors–related policy literature objectively [37].

This study used Tsinghua University’s policy analysis system, “ipolicy,” to retrieve policies. “ipolicy” is a unique characteristic analysis system, which can perform the specified analysis function according to users’ needs at different levels. At present, there are approximately 1,200,000 policy texts from 1949 to 2019 (including all the organs of the State Council, other central bodies, the National People’s Congress, and more than 34 provinces), which are still being updated [38].

In the retrieval module, the system provides various retrieval modes, and the content of the retrieval can be selected as full text or title retrieval. Advanced retrieval and retrieval in the results are also available to facilitate the user to find the text more accurately.

**Figure 1:**
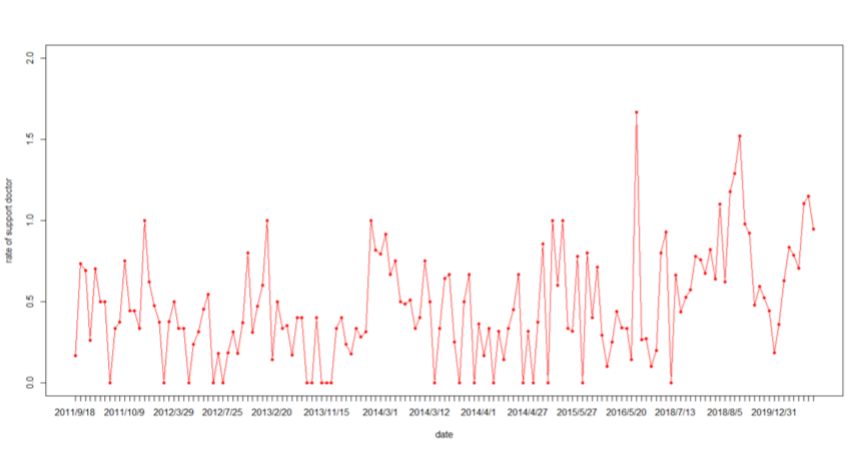
The rate of support doctors at the 151 time points from 2011–2020.

We used interrupted time series analysis (ITSA), a kind of longitudinal quasi-experimental design [39,40]. ITSA provides a segmented linear regression model, which could assess the effect of each central policy on changes in the “support doctor” and “blame doctor” online public opinion before the policy promulgation date and at 30 time points after the promulgation date. We combined some of the rates of sentimental categories to understand netizens’ (Internet Citizens [41]) attitudes toward violence against doctors events. Because the meanings of “blame doctor” and “support patient” are similar, we combined the 2 categories of results into the new category of “blame doctor.” Likewise, we combined the results of “blame patient” and “support doctor” into the new category of “support doctor.” Originally, we crawled 19,220 comments. For the sake of ITSA, we averaged the sentiments of comments of the same date. For those with only 1 comment on each day, we framed adjected comments of 3-4 days and selected the second date to represent the time point. Finally, we obtained 151 time points representing the online public opinion change (Figure 1). As the policy promulgation date overlapped with the 151 points, we finally obtained 15 policy intervention dates for ITSA. The models quantify both a level and slope change following the intervention, while accounting for the autocorrelation of rates. Autoregressive integrated moving average models were used to adjust for residual autocorrelation. We prepared the data with MS Excel, version 2019, and completed all statistical analyses with R statistical software package, version 4.0.2 (R Foundation for Statistical Computing), using a Type 1 error rate of .05 as the threshold for statistical significance.

The regression model used to fit these data is straightforward:

outcome_jt_ = β_0_ + β_1_ × time_t_ + β2 × level_j_+β3 × trend_jt_+ε_jt_

In this specific example (using the variable names from Table 7), the model is:

sentiment rate_t_ = β_0_ + β_1_ × time_t_ + β2 × policy_j_ + β3 × time after policy_jt_ + ε_jt_

## Results

### Number and Percentage of Coding Categories

Table 4 lists the preliminary results of coding, including the number of comments belonging to each category in the years 2011-2020, and the percentage of total comments in this period.

**Table 4:** Number and percentage of coding categories.

Coding category	Year								
	2011 (N=255)	2012 (N=1245)	2013 (N=232)	2014 (N=1473)	2015 (N=889)	2016 (N=1003)	2018^a^ (N=924)	2019^a^ (N=721)	2020^a^ (N=521)
Blame Big System, n (%)	30 (11.8)	132 (10.6)	25 (10.8)	174 (11.8)	58 (6.5)	74 (7.3)	(1.11)	(1.32)	(3.25)
Blame Medical System, n (%)	8 (3.1)	79 (6.3)	24 (10.3)	36 (2.4)	41 (4.6)	70 (6.9)	(0.34)	(1.2)	(2.19)
Blame Doctor, n (%)	85 (33.3)	312 (25.1)	91 (39.2)	191 (12.9)	183 (20.6)	133 (13.2)	(2.54)	(1.85)	(4.86)
Blame Patient, n (%)	68 (26.7)	167 (13.4)	24 (10.3)	362 (24.6)	161 (18.1)	207 (20.6)	(4.08)	(2.34)	(12.71)
Blame Other, n (%)	4 (1.6)	13 (1.0)	3 (1.3)	47 (3.2)	24 (2.7)	24 (2.3)	(0.0)	(0.56)	(1.92)
Support Doctor, n (%)	65 (25.5)	210 (16.9)	46 (19.8)	264 (17.9)	113 (12.7)	340 (33.9)	(2)	(1.95)	(3.83)
Support Patient, n (%)	60 (23.5)	71 (5.7)	15 (6.5)	19 (1.3)	44 (4.9)	8 (0.8)	(0.53)	(0.92)	(2.52)
Support Other, n (%)	0 (0.0)	1 (0.1)	1 (0.4)	1 (0.1)	2 (0.2)	0 (0.0)	0 (0.0)	(0.55)	(1.92)
N/A, n (%)	25 (9.8)	443 (35.6)	47 (20.3)	562 (38.2)	391 (43.9)	461 (45.9)	(0.82)	(1.77)	(3.72)

^a^Because the rate of 2017-2020 was from data mining, n is not available.

**Figure 2:**
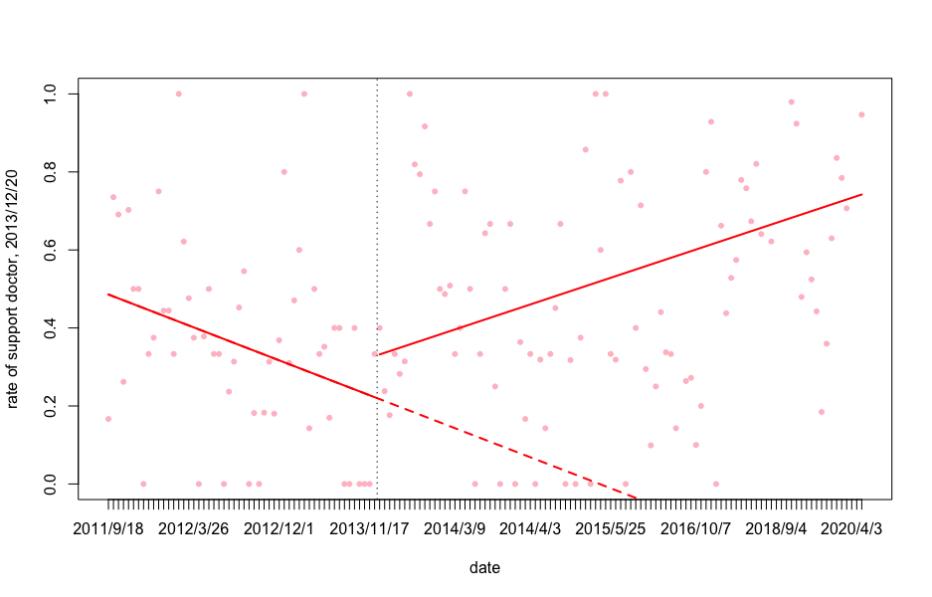
“Support doctor” proportion of public online opinion over time. Trend lines show slope change after lagged intervention date. Vertical dash line shows lagged intervention date of policy 2, Dec 20, 2013.

**Figure 3:**
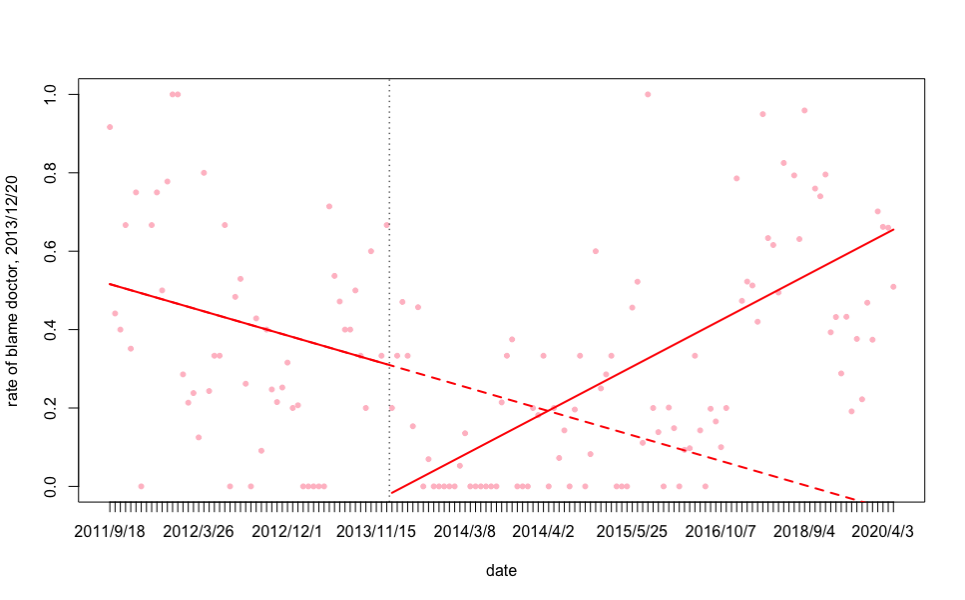
“Blame doctor” proportion of public online opinion over time. Trend lines show slope change after lagged intervention date. Vertical dash line shows lagged intervention date of policy 2, Dec 20, 2013.

As shown in Table 4, the proportion of “blame big system” is more stable than other categories; “blame doctor” peaked at 39.2% (91/232) in 2013, but decreased to 12.97% (191/1473) in 2014 and 4.86% in 2020; “support doctor” decreased from 2011 to 2012, but rose significantly from 2015 to 2020; “blame patients” reached the minimum of 10.3% (24/232) in 2013 and the maximum of 24.58% (362/1473) in 2014; “support patient” showed a downward trend, from the overall in 2020, when it was only 2.52% (Figures 2 and 3).

### Retrieval Results From the “ipolicy” System

The keywords we used to retrieve related policies were “doctor-patient,” “medical,” “violence,” “medical violence,” and “violence against doctors.” We then obtained related policies and regulations from 2011 to 2016 in China (Tables 5 and 6).

**Table 5:** Retrieval results from the “ipolicy” system.

Keywords	Results
Medical violence (医疗暴力)	Not related to the topic
Doctor-patient (医患)	Not related to the topic
Medical + violence (医疗或暴力)	Not related to the topic
Hurt doctors (伤医)	Two related entries in total
Violence against doctors (医暴)	Ninety-seven related entries in total

**Table 6:** Policy entries and netizens’ attitude trend.

Year	Policy entries, n			Netizens’ attitude trend, n	
	Total	Central policies	Local policies	Blame doctor	Support doctor
2011	12	0	19	—^a^	—
2012	24	11	12	↓^b^	↓
2013	27	16	11	↑^c^	—
2014	27	12	13	↓	↑
2015	4	4	0	↑	↓
2016	2	2	0	↓	↑
2017	3	3	0	↓	↓
2018	0	0	0	↓	↓
2019	0	0	0	—^d^	—
Sum	99	48	55		

^a^No change in attitude.

^b^Decrease in ratio of related attitude.

^c^Increase in ratio of related attitude compared with last year.

^d^Not available.

We browsed these 112 policies, and 19 policies were promulgated by the central government (Multimedia Appendix 3), of which the most relevant central policy documents are as follows:

1. Severe punishment of any illegal activities that would harm the safety of doctors or patients. We will severely punish illegal activities that disrupt regular medical order and improve the ability to solve medical disputes [15].
2. Keep improving awareness of and fight against illegal medical activities and crimes [23].
3. Resolutely combat crimes involving hospitals to maintain order and to punish mob behavior, etc. [24].
4. Establish a medical risk-sharing mechanism with medical liability insurance as the main form, and have this mechanism play an important role in medical dispute resolution and medical risk management [12].

As shown in Tables 7 and 8, for the first central policy (promulgated on October 26, 2012), at baseline (before the policy was promulgated), there was neither a significant level nor trend change in the rate of “support doctor.” However, there was a drop of 2% per time point in the “blame doctor” trend (Figures 2 and 3).

There was an increase in the “support doctor” trend after each policy promulgation (average trend changes of 0.011 of the policies 2-12, *P*<.05), but most of the slope change was not significant. At almost every time point in the policy promulgation, there was a decline in the proportion of “blame doctor” (average level drop of –0.784 of the policies 2-4, *P*<.05); however, there was also a trend change lag (average slope change of 0.018 of the policies 2-12, *P*<.05).

**Table 7:** Support doctor regression parameters at 15 policy time points.

Policy	Policy date	β1	*P*	β2	*P*	β3	*P*
1	October 26, 2012	–.009	.235	.035	.825	.013	.097
2	December 20, 2013	–.005	.124	.106	.391	.009	.010
3	January 29, 2014	–.005	.176	.126	.365	.009	.023
4	March 26, 2014	–.005	<.001	–.165	.034	.016	<.001
5	April 22, 2014	.001	.789	–.125	.169	.010	<.001
6	May 8, 2014	.001	.881	–.041	.679	.010	<.001
7	July 9, 2014	.001	.717	.005	.958	.010	<.001
8	September 4, 2014	.001	.943	–.064	.519	.011	<.001
9	October 27, 2014	.001	.660	–.104	.318	.012	<.001
10	June 24, 2015	.001	.688	.007	.929	.011	<.001
11	March 24, 2016	.001	.646	–.041	.708	.013	<.001
12	May 13, 2016	.001	.863	.067	.553	.011	.007
13	June 26, 2017	.001	<.001	.123	.272	.013	.066
14	July 31, 2018	.001	.081	.441	.025	.005	.781
15	March 5, 2019	.002	.109	.120	.587	.006	.766

**Table 8:** Blame doctor regression parameters at 15 policy time points.

Policy	Policy date	β1	*P*	β2	*P*	β3	*P*
1	October 26, 2012	–.017	.057	–.038	.8316	.020	.031
2	December 20, 2013	–.004	.2478	–.335	.007	.011	.002
3	January 29, 2014	–.005	.1259	–.277	.046	.012	.002
4	March 26, 2014	–.004	<.001	–.172	<.001	.015	<.001
5	April 22, 2014	–.005	<.001	.025	.4097	.016	<.001
6	May 8, 2014	–.005	<.001	.103	.001	.016	<.001
7	July 9, 2014	–.005	<.001	.128	<.001	.015	<.001
8	September 4, 2014	–.005	<.001	.151	<.001	.015	<.001
9	October 27, 2014	–.005	<.001	.256	<.001	.013	<.001
10	June 24, 2015	–.004	<.001	.332	<.001	.011	<.001
11	March 24, 2016	–.004	<.001	.435	<.001	.008	<.001
12	May 13, 2016	–.004	<.001	.398	<.001	.010	.018
13	June 26, 2017	–.002	.062	.358	.038	.004	.646
14	July 31, 2018	.000	.898	.120	.562	–.002	.937
15	March 5, 2019	.001	.716	–.251	.228	.008	.7322

## Discussion

### Principal Findings

In 2017, discussion in *The Lancet* suggested that recommendations to rebuild patient–physician trust in China should not only focus on the physician’s side but also on the patient’s side [42,43]. Recently, particularly during COVID-19, voices to stop attacks against health care personnel have been raised worldwide [44-46]. All the health professional associations, societies, and organizations from all specialties and disciplines should unite to protect staff, while the government should play a major role by enacting strong and appropriate policies [47].

The current research has found that the 14 violence against doctors–related policies promulgated by the Chinese central government are associated with the sentiment expressed in online public opinions. The rate of online comments supporting doctors tends to increase significantly (see Table 7 for significant values) after almost each policy promulgation, while the rate of blaming doctors declined at each policy promulgation time point. However, the trend of blaming doctors then rises later after every policy is enacted. The overall declining trend of blaming doctors (39.22% in 2013 versus 4.86% in 2020) and the increased ITS trend for each policy time point imply that policy effects are only temporary. Particularly, in recent years (2017-2020), there have been few polices, and the ITS trends are not significant. It is time to reflect on the effects of policies.

The recent discussions regarding the relationship between violence against doctors events and health care insurance are very lively. In 2018, China’s Congress promulgated the Social Insurance Law of the People’s Republic of China (2018 Amendment) [48]. Although China’s medical security system is continually improving, there are still some problems. Furthermore, the severe current aging of our society and young people’s increasing work pressure have led to an increase in the incidence of some major diseases. For some significant diseases, many drugs are not included in the medical insurance coverage, and patients must bear most or all of the medical expenses alone. Patients are likely to experience significant imbalances, and it is easy to dissolve this inconsistency. The balance is blamed on the medical institution and the most directly contacted population—medical staff [49-51]. Our study has provided this new patient viewpoint from the health and science education for the public. In this study, we focused on netizens’ comments on major violence against doctors events and their attitudes toward doctors, patients, and the government as direct evidence to understand public opinions and the state of the physician–patient relationship. We found a significant relationship (see Tables 7 and 8 for significant *P-*values) between the time of implementation of government health reform policies and the shift of public opinion from “blame doctor” to “support doctor.” In 2011 and 2012, the proportion of “blame doctor” and “support doctor” opinions were relatively balanced. However, in 2013, the proportions began to shift: the proportion of “blame doctor” rose while “support doctor” dropped. Because of this differentiation, the government had to adopt some policies. By the end of 2013, the public mood had eased up after the implementation of related health policies. In 2014, the number of related health policies peaked but then decreased in 2015. Thus, the proportion of “support doctor” opinions fell in 2015. Again, the situation improved after the enactment of central regulations in 2016 (Figures 2 and 3).

From the “ipolicy” system, we retrieved related policies and regulations from 2011 to 2016 in China and found the most relevant central policies to be those decreeing stricter enforcement and severe punishment for those endangering the safety of doctors and other medical staff [27]. Furthermore, in 2014 and 2016, further policies showed more awareness of fighting illegal medical activities [23] and concerns for resolving mob-related criminal behavior at medical hospitals [24]. Before these enactments, the enforcement of punishment for violence against doctors and the level of security for hospital staff were weak, allowing doctors and other medical staff to become victims [52].

Nonetheless, it is the role of the government to enforce proper regulations to prevent danger to medical staff. Our results show that the only interaction between attitude and turning point occurred from 2013 to 2014, which is connected with relevant policies between 2014 and 2016 contributing to a significant drop in citizens’ “blame” of doctors (see Table 8 for significant *P* values). These efforts show that the Chinese government has heightened vigilance toward the problems displayed by increased violence against doctors incidents and more order in controlling the harms caused by such incidents. However, their efforts remain slightly below the US approach, suggesting that the Chinese government should continue to adopt more control and management roles in medical emergencies. According to the Chinese government, “Following the Occupational Safety and Health Act of 1970, 26 US states and two US territories require employers to develop detailed violence prevention programs. The Occupational Safety and Health Administration (OSHA) have also increased the number of visits to medical facilities, from 11 in 2010 to 86 in 2014. The American Government Accountability Office (GAO) gave a specific recommendation in its 2016 Work Report. Employers should be punished if they allow employees to be exposed to potential workplace violence, especially if they have a history of violence, assault or physical assault; provide additional information to inspectors in specific court summon cases to help develop a system of summonses; follow up confirmation of the need for further measures” [42,43,53].

In analyzing the comments rather than the news articles themselves, we provided a more direct measure of the public’s perspective and attitude toward the health care sphere and their stance on the patient–physician bond. The data we collected on these comments can further be used as a potential indicator of health care quality, as few reliable measures exist. Wang et al [54] utilized medical malpractice incidents as a probable marker of health care quality, but a more robust and direct measure is needed to probe the difficulties experienced by the Chinese medical field. We analyzed the comments based on the variable of time, so we can see changes and patterns through time, compared to previous studies, which examined more qualitative methods to seek reliable measures of the patient–doctor relationship [55,56]. Comments made by the general public can reveal their emotions and opinions, as well as their perceptions and viewpoints on the shortcomings of the health care field and expose needed reforms for the improvement of trust in medical professionals.

Our study also contributes to the qualitative study of feelings of mistrust in the physician–patient relationship in China. A survey of 107 health care physicians in Guangdong province, China, reported that physicians are caught in a “vicious cycle of mistrust” due to various contributing factors of work overload, conflicts of interest, unreasonable requests of patients, and more [57]. Restoring public trust in their physicians requires a strong foundation supported by government regulations for effective delivery and more transparent methods of health care quality assessments.

The media play an essential role in contributing to overall public attitudes toward the physician–patient relationship and overall health care system when it documents violence against doctors events for citizens. Both print and electronic media have a responsibility not to sensationalize the news. Health care workers and officials have cited negative media coverage and sensationalism of violence against doctors events as a prime reason contributing to the deteriorating doctor–patient relationship and mistrust in the medical field [58]. The practice of medicine is highly complex. Diagnosis of a patient is an essentially hypothetico-deductive process, and with new evidence presented through investigations and knowledge, this process continues to be refined. However, whatever the diagnosis may be, patient management generally considers such uncertainties and treatment continues. One of the purposes of this paper has been to analyze whether the government’s policies can affect public opinion on doctor–patient issues, mainly regarding the policies issued by the government on how to report violence against doctors events to influence public opinion. We expect that government policy could intervene in doctor–patient relationships through medical information and media, and our data demonstrate this. However, we do not expect that the decrease in netizens’ blame of doctors and the increase in support for doctors will immediately end the trend of violent acts against doctors. Another purpose of our study was to understand the current trends in the medical network for violence against doctors and hope to find a breakthrough to ease public opinion. Although the media is not the initiator of such medical violence incidents, they are a vital participant in the aftermath, playing the critical role of disseminator of information. Thus, information authenticity and other aspects need to be considered.

In China, the internet environment is managed and controlled by the government, which pays attention to the impact of the network on society. If news media views drive the mood of internet users, then internet users’ irrational speech will increase, also causing more extreme emotions and comments (broken window effect). Media reports tend to play an essential role in driving social norms, which influence people’s behavior and outlooks on topics [57]. To prevent the news of medical violence harming public opinion, network administrators may need to carry out monitoring measures. In our study, we observed numerous emotionally charged comments, which indicate that these news media network comments have not been filtered by management; otherwise, there would be fewer “blame-style” comments. However, measuring the extent of comment monitoring of online news platforms is beyond the scope of our study. When an outlet is provided for netizens to discuss current events, express their views, and vent their emotions, it should guarantee as much free speech as possible, but it is unclear whether the same should apply in terms of medical violence.

China’s intentional homicide has increased in our statistical data years—eg, from 10,285 in 2013 to 17,946 in 2015 [59]. The killing of a doctor is intentional homicide and with the increase in this crime, there has been an increase in the number of doctors killed. However, this does not mean that the relationship between doctors and patients has deteriorated further. The current research focuses on violence against doctors, not just homicides. According to statistics on other similar topics, the incidence of violent medical injuries in China has declined since 2015 [60].

Our study has several limitations. Our scope is limited to the violence against doctors events we selected for the study and to comments referring to those incidents, but we did not cover all types of medical incidents. Furthermore, the sample size, timeline, and number of comments are limited for objective reasons. Hence, we expect further research to be conducted to improve the study of people’s emotional valence in violence against doctors events. In future research, we hope to study the key factors influencing people’s emotions in violence against doctors events through randomized controlled trials. Future studies could also compare violence against doctors events across hospital types, for example, comparing violence against doctors events in private versus public hospitals. Examinations of violence against doctors events across departments within hospitals could also offer information on variations across specialties (eg, obstetrics, pediatrics, and other departments). Regional variations could be another area of inquiry. We seek to provide practical evidence and interventions to reduce the incidence of violence against doctors events and improve the doctor–patient relationship in China.

The causes of the rise and severity of violence against doctors in China are numerous and can be traced back to the systemic roots of inadequate investment in the health care system with subpar training and salaries for physicians, leading to higher chances of medical errors, administrative exploitation, and ineffective communication between patients and medical staff. Other factors may be cultural and societal, such as unfavorable and sensationalized news articles about doctors, the public’s limited knowledge of medicine, unrealistic expectations from doctors and treatments, and detrimental out-of-pocket expenses for patients. As such, the impact of violence against doctors is of great concern and should not be taken lightly for the future of Chinese medicine. Further studies highlighting the role of the government and media should be conducted to improve the physician–patient relationship.

### Conclusions

By matching the policy documents for medical violence with the online commentary attitudes, we found that the state’s administrative intervention effectively guided public opinion. Notably, these findings provide scientific evidence and support for the government’s role in intervention and prevention of both cyber and physical violence.

During the COVID-19 pandemic, many countries have been advocating home isolation, leading to a major increase in people’s reliance on the internet for social support and health care information. During the pandemic, a harmonious patient–physician relationship has become an important guarantee for countries in responding to the epidemic. The results of this study show that the government’s reasonable and effective policy guidance for violence against doctors has a significant impact (see Tables 7 and 8 for significant values) on the public’s comments on online media reports, which in turn contribute to the normal medical order as well as epidemic prevention and control.

## Appendix

Multimedia Appendix 1 Comments acquisition.

Multimedia Appendix 2 Details of the VAD cases reported by international media from 2011–2020.

Multimedia Appendix 3 Nineteen policies promulgated by the Chinese central government.

## Abbreviations

ITSA: interrupted time series analysis

## References

[1] Liew L (1997). The Chinese Economy in Transition: From Plan to Market.

[2] Xi J (2017). Full text of Xi Jinping's report at 19th CPC National Congress. China Daily.

[3] Hesketh T, Wu D, Mao L, Ma N (2012). Violence against doctors in China. BMJ.

[4] Pan J, Liu D, Ali S (2015). Patient dissatisfaction in China: What matters. Soc Sci Med.

[5] The Lancet (2012). Ending violence against doctors in China. The Lancet.

[6] Lupton D, McLean J (1998). Representing doctors: discourses and images in the Australian press. Soc Sci Med.

[7] Happer C, Philo G (2013). The Role of the Media in the Construction of Public Belief and Social Change. J. Soc. Polit. Psych.

[8] Rippon TJ (2000). Aggression and violence in health care professions. J Adv Nurs.

[9] Mouhssine E, Khalid C (2018). Social Big Data Mining Framework for extremist content detection in Social Networks.

[10] Chen H (2008). Sentimentaffect analysis of dark web forums: Measuring radicalization on the internet. Yang CC, Chen WH, Hsu WL, Wu TC, Zeng D. eds.

[11] Ministry of Justice of the People's Republic of China (2020). Criminal Law of the People's Republic of China.

[12] National Health and Family Planning Commission (2015). National Opinions of the National Health and Family Planning Commission, the Ministry of Justice, the Ministry of Finance, and their Departments on Strengthening the Work of Medical Liability Insurance.

[13] National Health Commission of the People's Republic of China (2013). A circular on the issuance of a special action plan to maintain medical order and crack down on crimes related to medical treatment.

[14] Tang J, Kuang C (2020). Can Yinao be stopped by the security hospital? Today's Morning Post (in Chinese).

[15] National Health Commission of the People's Republic of China (2020). Notice on the issuance of a special action plan to maintain medical order and crack down on medical crimes [Article 43].

[16] Baker P (1994). Illness meanings and perceptions of control and uncertainty in women with breast cancer. Master Thesis.

[17] Ko N, Hsu S (2005). Informational needs, health locus of control and uncertainty among women hospitalized with gynecological diseases. Chang Gung Med J.

[18] Leotti LA, Iyengar SS, Ochsner KN (2010). Born to choose: the origins and value of the need for control. Trends Cogn Sci.

[19] Averill JR (1973). Personal control over aversive stimuli and its relationship to stress. Psychological Bulletin.

[20] Kay AC, Gaucher D, Napier JL, Callan MJ, Laurin K (2008). God and the government: Testing a compensatory control mechanism for the support of external systems. Journal of Personality and Social Psychology.

[21] Zhang W, Deng Z, Hong Z, Evans R, Ma J, Zhang H (2018). Unhappy Patients Are Not Alike: Content Analysis of the Negative Comments from China's Good Doctor Website. J Med Internet Res.

[22] Yang Q, Pan J (2017). Control under times of uncertainty: the relationship between hospital competition and physician-patient disputes. Int J Equity Health.

[23] National Health Commission of the People's Republic of China (2014). The Comprehensive Treatment of Social Management Committee. Notice of the Ministry of Public Security on deepening the establishment of ping an hospital to further crack down on doctor-related crimes and maintain normal medical order.

[24] National Health Commission of the People's Republic of China, The Comprehensive Treatment of Social Management Committee, The Ministry of Public Security of the People's Republic of China (2016). Notice from the Ministry of Justice on further efforts to maintain medical order.

[25] Liu Z (2019). New Media Influence Index Report.

[26] Kantar (2016). China's Social Media Influence Rankings 2016.

[27] Kanter (2016). Kantar China Media Impact Report.

[28] Wang L, Jiang X, Zhang S, He L, Peng L (2016). Statistical analysis of medical damage liability dispute cases in China. Chin J Health Stats.

[29] Tencent News.

[30] Sohu News.

[31] iFeng News.

[32] Sina News.

[33] Tianya Forum.

[34] NetEase News.

[35] Davey K, Ravishankar V, Mehta N, Ahluwalia T, Blanchard J, Smith J, Douglass K (2020). A qualitative study of workplace violence among healthcare providers in emergency departments in India. Int J Emerg Med.

[36] Vora P (2017). India's hospital assaults: patients blame doctors while doctors blame the broken health system. Scroll.in.

[37] Cui H, Tao R, Jian Z (2015). Quantitative Research on Policy Literature: The New Direction of Public Policy Research. J Public Manag.

[38] Ipolicy Policy Analysis System (2017). Center for Government Literature.

[39] Giuliani ME, Liu G, Xu W, Dirlea M, Selby P, Papadakos J, Abdelmutti N, Yang D, Eng L, Goldstein DP, Jones JM (2019). Implementation of a Novel Electronic Patient-Directed Smoking Cessation Platform for Cancer Patients: Interrupted Time Series Analysis. J Med Internet Res.

[40] Choi S, Yuen HM, Annan R, Pickup T, Pulman A, Monroy-Valle M, Aduku NEL, Kyei-Boateng S, Velásquez Monzón CI, Portillo Sermeño CE, Penn A, Ashworth A, Jackson AA (2018). Effectiveness of the Malnutrition eLearning Course for Global Capacity Building in the Management of Malnutrition: Cross-Country Interrupted Time-Series Study. J Med Internet Res.

[41] Xu Y (2012). Understanding netizen discourse in China: formation, genres, and values. China Media Res.

[42] Lyu Z, Wu S, Cai Z, Guan X (2016). Patient–physician trust in China: health education for the public. The Lancet.

[43] Tucker JD, Wong B, Nie J, Kleinman A (2016). Rebuilding patient–physician trust in China. The Lancet.

[44] McKay D, Heisler M, Mishori R, Catton H, Kloiber O (2020). Attacks against health-care personnel must stop, especially as the world fights COVID-19. The Lancet.

[45] Dzau VJ, Kirch D, Nasca T (2020). Preventing a Parallel Pandemic — A National Strategy to Protect Clinicians’ Well-Being. N Engl J Med.

[46] Zhang Z, Liu S, Xiang M, Li S, Zhao D, Huang C (2020). Protecting healthcare personnel from 2019-nCoV infection risks: lessons and suggestions. Frontiers of Medicine.

[47] Zhang X, Guo X, Lai K, Yi W (2018). How does online interactional unfairness matter for patient–doctor relationship quality in online health consultation? The contingencies of professional seniority and disease severity. European Journal of Information Systems.

[48] Standing Committee of National People's Congress (2018). Social Insurance Law of the People's Republic of China (2018 Amended).

[49] Zhu K, Zhang L, Yuan S, Zhang X, Zhang Z (2017). Health financing and integration of urban and rural residents’ basic medical insurance systems in China. Int J Equity Health.

[50] Zhang J, Cai Y (2018). Medical disputes and mediation in China: Government and responsibility shifting. China Information.

[51] An P, Ye Y, Li Q, Liu B, Lian K, Yin J, Hao J, Zhou S, Gan L (2020). Medical disputes in relation to prenatal ultrasound in China. Ultrasound Obstet Gynecol.

[52] Peng W, Ding G, Tang Q, Xu L (2016). Continuing violence against medical personnel in China: A flagrant violation of Chinese law. Bioscience trends.

[53] Wyatt R, Anderson-Drevs K, Van Male LM (2016). Workplace Violence in Health Care. JAMA.

[54] Wang Z, Li N, Jiang M, Dear K, Hsieh C (2017). Records of medical malpractice litigation: a potential indicator of health-care quality in China. Bulletin of the World Health Organization.

[55] Liu Z, Zhang Y, Asante JO, Huang Y, Wang X, Chen L (2018). Characteristics of medical disputes arising from dental practice in Guangzhou, China: an observational study. BMJ Open.

[56] Nie J, Li L, Gillett G, Tucker JD, Kleinman A (2017). The crisis of patient-physician trust and bioethics: lessons and inspirations from China. Developing World Bioeth.

[57] Wilson JQ, George LK (1982). Broken Windows: the police and neighborhood safety. The Atlantic Monthly.

[58] Nie J, Cheng Y, Zou X, Gong N, Tucker JD, Wong B, Kleinman A (2017). The vicious circle of patient-physician mistrust in China: health professionals’ perspectives, institutional conflict of interest, and building trust through medical professionalism. Developing World Bioeth.

[59] Fu G, Ma L (2017). Serious Violent Crimes and The Social Contradictions from the Perspective of Judicial Data. Journal of Law Application:Judicial Case (in Chinese).

[60] Mei S, Li Z, Zhang X (2019). Qualitative Data Analysis of 228 Cases of Workplace Violence on Medical Staffs Based on Internet Media. Chin Health Serv Manag (in Chinese).

