# Supplementary material for: Measuring Public Reaction to Violence Against Doctors in China: Interrupted Time Series Analysis of Media Reports
Source: J Med Internet Res. 2021 Feb 16;23(2):e19651. doi: 10.2196/19651 (PMC7925148; doi:10.2196/19651)
Supplement: Multimedia Appendix 1 [file jmir_v23i2e19651_app1.docx]

Appendix A

Comments Acquisition

Python 2.7.12 programming language was adopted to write the software application for collecting the comments. We then sampled approximately 400 comments for each VAD news report from the news sources as mentioned in Table 4. The main technical steps were:

1. Collect the URL from these sources.

2. Acquire the web pages located by all URLs with the software Python library Requests 2.17.3.

3. Use the standard Regularization Expression library and Beautiful Soup 4.4.0 toolboxes that come with Python, as well as an XML parsing library of Python to extract comments from the web pages.

4. Openpyxl 2.4.8 (a Python library for handling Excel files) and Pandas 0.20.1 (a Python library for data analysis) were applied to store comments in Microsoft Excel for coding.
