# Supplementary material for: Measuring Public Reaction to Violence Against Doctors in China: Interrupted Time Series Analysis of Media Reports
Source: J Med Internet Res. 2021 Feb 16;23(2):e19651. doi: 10.2196/19651 (PMC7925148; doi:10.2196/19651)
Supplement: Multimedia Appendix 2 [file jmir_v23i2e19651_app2.docx]

Appendix B

Details of the VAD cases reported by international media from 2011–2020

|  | time | victim title | outcome |  | murderer’s corresponding doctor | murderer’s profession | disease of the murderer/relatives | hospital city | hospital level | hospital property |
| --- | --- | --- | --- | --- | --- | --- | --- | --- | --- | --- |
| 1 | 2011年9月15日 | Chief physician | death | Otolaryngology | Yes | teacher | Laryngeal cancer | Beijing | Grade III class A | public |
| 2 | 2012年3月25日 | Graduate student | death | Rheumatology | No | adolescent | Ankylosing spondylitis | Harbin, Heilongjiang | Grade III class A | public |
| 3 | 2012年11月29日 | Chief physician | death | Acupuncture | No | retired | Cerebral infarction | Tianjin | Grade III class A | public |
| 4 | 2013年10月25日 | Associate chief physician | death | Otolaryngology | No | unemployed | Empty nose syndrome | Wenlin, Zhejiang | Grade III class B | public |
| 5 | 2014年2月25日 | Nurse | spinal cord concussion | Emergency | Yes | officials | inpatient | Nanjing, Jiangsu | Grade III class A | public |
| 6 | 2014年3月24日 | Associate chief physician | suicide | Oncology | Yes | Not reported | cancer | Shanghai | Grade III class A | public |
| 7 | 2015年7月16日 | Chief physician | seriously injured | Neurology | Yes | unemployed | headache | Longmen, Guangdong | Grade II class A | public |
| 8 | 2015年1月24日 | Doctor on duty | death | Orthopedic | No | citizen | drunk | Luoyang, Henan | Grade II class A | public |
| 9 | 2015年7月18日 | Chief physician | joint support | N/A | N/A | N/A | N/A | Beijing and Shanghai | Grade III class A | from both |
| 10 | 2014年2月18日 | Physician | seriously injured | General Surgery | No | farmer | hernia | Baoding, Hebei | Grade II class B | public |
| 11 | 2016年5月18日 | Physician | death | Ophthalmology and Otorhinolaryngology | Yes | Not reported | child traffic injury | Shaodong, Hunan | Grade II class A | public |
| 12 | 2016年5月7日 | Chief physician | death | Stomatology | Yes | unemployed | tooth discoloration | Guangzhou, Guangdong | Grade III class A | public |
| 13 | 2016年10月3日 | Attending physician | death | Pediatrics | Yes | farmer | daughter fatal fever | Laiwu, Shandong | Grade III class B | transferred to public in 2018 |
| 14 | 2018年3月14日 | Chief physician | death | Gastroenterology | Yes | Repeat offenders | wife colonoscopy for anesthesia | Xuancheng, Anhui | Grade II class A | public |
| 15 | 2018年7月16日 | Attending physician | death | Gastroenterology | No | Not reported | Not reported | Tianjin | Grade III class A | public |
| 16 | 2019年10月22日 | Associate chief physician | death | Anorectal | Yes | Not reported | Colorectal cancer | Yinchuan, Gansu | Grade III class A | public |
| 17 | 2019年12月24日 | Associate chief physician | death | Emergency | Yes | Not reported | Cerebral infarction | Beijing | Grade III c  lass B | public |

Note: Case 9 is 17 famous doctors’ joint support for the victim physician in the case.
