# Supplementary material for: Measuring Public Reaction to Violence Against Doctors in China: Interrupted Time Series Analysis of Media Reports
Source: J Med Internet Res. 2021 Feb 16;23(2):e19651. doi: 10.2196/19651 (PMC7925148; doi:10.2196/19651)
Supplement: Multimedia Appendix 3 [file jmir_v23i2e19651_app3.docx]

Appendix C

| Policy No. | Policy Date | | Title | Promulgation Date | Publishing department | aim |
| --- | --- | --- | --- | --- | --- | --- |
| 1 | 1 | | Mental Health Law of the People’s Republic of China | October 26, 2012 | NPC Standing Committee | regular patient |
| 2 | 2 | | Notice on Issuing the Special Action Plan for Maintaining Medical Order and Cracking down on Medical-related Crimes | December 20, 2013 | National Health and Family Planning | regular both |
| 3 | 3 | Notice of the General Office of the National Health and Family Planning Commission on Carrying out the Work of Signing the Agreement on Non-acceptance and Non-send “Red Envelopes” by the Doctors and Patients | | January 29, 2014 | National Health and Family Planning | regular both |
| 4 | 4 | Notice on Issuing the Opinions on Promoting the Comprehensive Reform of County-level Public Hospitals | | March 26, 2014 | National Health and Family Planning | regular doctor |
| 5 | 5 | The Supreme People’s Court, the Supreme People’s Procuratorate, the Ministry of Public Security, etc. issued the “Opinions on Punishing Medical-related Illegal Crimes and Maintaining Normal Medical Order” Notice | | April 22, 2014 | Supreme People’s Court, Supreme People’s Procuratorate, Ministry of Public Security | regular patient |
| 6 | 6 | Notice of the National Health and Family Planning Commission on the Public Consultation on the “Measures for Medical Quality Management (Draft for Comment)” | | May 8, 2014 | National Health and Family Planning | regular doctor |
| 7 | 7 | Opinions of the National Health and Family Planning Commission, the Ministry of Justice, the Ministry of Finance and Other Departments on Strengthening the Work of Medical Liability Insurance | | July 9, 2014 | National Health and Family Planning | regular doctor |
| 8 | 8 | Notice on Deepening the Establishment of “Safe Hospitals” and Doing a Good Job in Fighting Medical-related Illegal Crimes and Maintaining Normal Medical Order | | September 4, 2014 | National Health and Family Planning | regular patient |
| 9 | 9 | Amendment to the Criminal Law of the People’s Republic of China (9) (Draft) provisions and draft descriptions | | October 27, 2014 | NPC Standing Committee | regular doctor |
| 10 | 9 | Announcement of the General Office of the National Health and Family Planning Commission on the work of further rectifying the medical order and combating illegal medical practice | | December 17, 2014 | National Health and Family Planning | regular doctor |
| 11 | 10 | Report by the Law Committee of the National People’s Congress on the revision of the “Amendment to the Criminal Law of the People’s Republic of China (9) (Draft)” | | June 24, 2015 | National People’s Representative Meeting | regular patient |
| 12 | 11 | Notice on further doing a good job in maintaining medical order | | March 24, 2016 | National Health and Family Planning | regular patient |
| 13 | 12 | Notice of National Health and Family Planning Commission, National Development and Reform Commission, Ministry of Education, etc. on Issuing Opinions on Strengthening the Reform and Development of Children’s Medical and Health Services | | May 13, 2016 | National Health and Family Planning Commission, National Development and Reform Commission (including former National Development Planning Commission and former National Planning Commission), Ministry of Education | regular both |
| 14 | 13 | Notice on Issuing Opinions on Strictly Preventing and Controlling Medical-related Illegal Crimes and Maintaining Normal Medical Order | | June 26, 2017 | National Health and Family Planning Commission, Ministry of Public Security, State Administration of Traditional Chinese Medicine | regular doctor |
| 15 | 14 | Regulation on the Prevention and Handling of Medical Disputes | | July 31, 2018 | State Council | regular doctor |
| 16 | 14 | Notice by the National Development and Reform Commission, the People’s Bank of China, the National Health Commission, and Other Departments of Issuing the Memorandum of Understanding on Taking Joint Disciplinary Actions against Persons Liable for Dishonest Acts that Seriously Disrupt the Normal Order of Medical Services | | September 25, 2018 | National Development and Reform Commission (including the former National Development Planning Commission and the former State Planning Commission), the People’s Bank of China, and the National Health Commission | regular patient |
| 17 | 14 | Notice by the Supreme People’s Court of Issuing the Work Priorities of People’s Courts in 2019 | | January 24, 2019 | Supreme People’s Court | regular patient |
| 18 | 14 | Notice of the General Office of the National Health Commission on Printing and Distributing the Hospital Smart Service Classification Evaluation Standard System (Trial) | | March 5, 2019 | National Health Commission | regular patient |
